# High maltose sensitivity of sweet taste receptors in the Japanese macaque (*Macaca fuscata*)

**DOI:** 10.1038/srep39352

**Published:** 2016-12-16

**Authors:** Emiko Nishi, Kei Tsutsui, Hiroo Imai

**Affiliations:** 1 Molecular Biology Section, Department of Cellular and Molecular Biology, Primate Research Institute, Kyoto University, 41-2 Kanrin, Inuyama, Aichi, Japan.

## Abstract

Taste sensitivity differs among animal species depending on feeding habitat. To humans, sucrose is one of the sweetest natural sugars, and this trait is expected to be similar in other primates. However, previous behavioral tests have shown that some primate species have equal preferences for maltose and sucrose. Because sweet tastes are recognized when compounds bind to the sweet taste receptor Tas1R2/Tas1R3, we evaluated the responses of human and Japanese macaque Tas1R2/Tas1R3 to various natural sugars using a heterologous expression system. Human Tas1R2/Tas1R3 showed high sensitivity to sucrose, as expected; however, Japanese macaque Tas1R2/Tas1R3 showed equally high sensitivity to maltose and sucrose. Furthermore, Japanese macaques showed equally high sensitivity to sucrose and maltose in a two-bottle behavioral experiment. These results indicate that Japanese macaques have high sensitivity to maltose, and this sensitivity is directly related to Tas1R2/Tas1R3 function. This is the first molecular biological evidence that for some primate species, sucrose is not the most preferable natural sugar, as it is for humans.

In animals, taste provides important information to evaluate the quality and quantity of nutrients and toxins in foods. Sweet, umami (savory), bitter, salty, and sour are referred to as the five basic tastes^1^. Sweet and umami, which indicate sugars and proteins, respectively, are preferred tastes. Because plants convert CO_2_ to starches by photosynthesis and transport or store this energy as various types of sugars, sweet taste is particularly important for the detection of energy sources in plants by many phytophagous and omnivorous animals, including primates. Sweet taste compounds are mainly detected by binding to the sweet taste receptors Tas1R2 and Tas1R3, which are expressed on the surface of the tongue^2^. After sweet taste is detected by Tas1R2/Tas1R3, the signal from Tas1R2/Tas1R3 is transmitted to the brain, enhancing appetite and feeding behaviors to promote calorie intake. The amino acid sequence of Tas1R2/Tas1R3 differs among species, and these differences may result in functional differences related to animal behavior. Based on a previous behavioral experiment, most primates, including humans, prefer the sweet taste of sucrose, but the ability to detect sweet tastes differs among species^3^. On the other hand, few plants store high concentrations of sucrose so it is not clear how this ability gives primates an advantage in nature.

Interestingly, it has been suggested that some Old World monkeys, such as macaques and baboons, show similar sensitivity to maltose and sucrose^3^. Because humans are much less sensitive to maltose than sucrose^4^,^5^, the mechanism underlying this interspecific difference is the subject of a long-standing debate. There may be an additional receptor specific to maltose; alternatively, differences in sensitivity may be explained by Tas1R2/Tas1R3 divergence. Here, we present molecular and behavioral evidence for the evolution of Tas1R2/Tas1R3 in primates, which adapted to low-sucrose foods in the wild.

## Results

**Figure 1:**
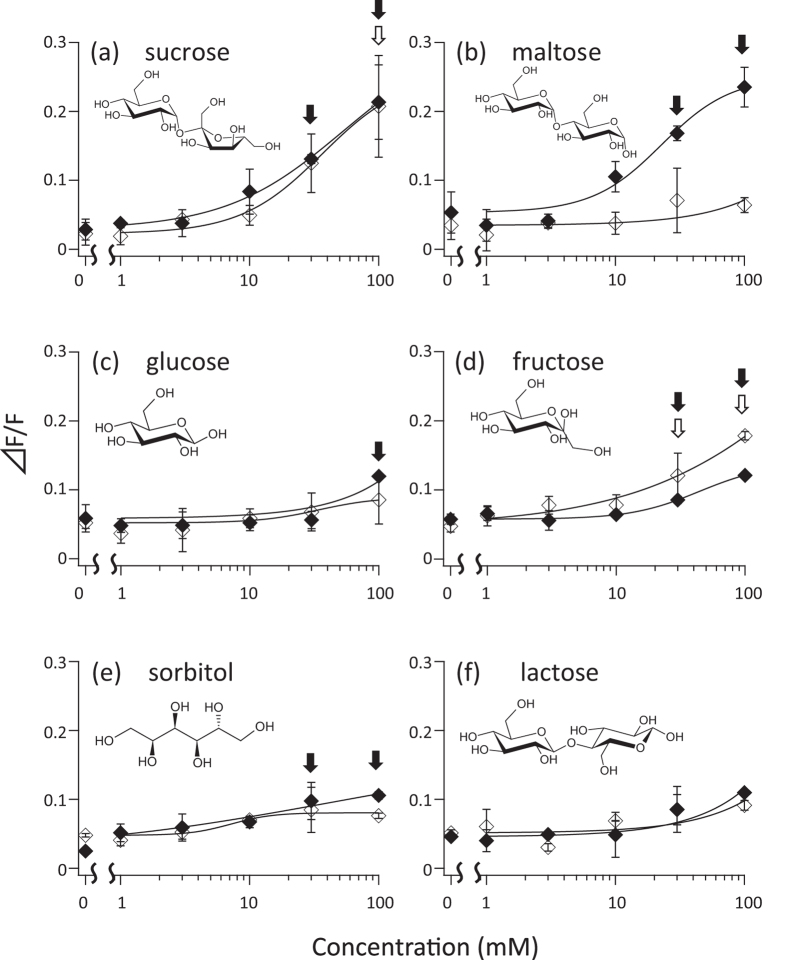
Tas1R2/Tas1R3 response to various natural sugars. Sucrose (**a**), maltose (**b**), glucose (**c**), fructose (**d**), sorbitol (**e**), and lactose (**f**) were added to Hs Tas1R2/Tas1R3 (□) and Mf Tas1R2/Tas1R3 (■). The structural formula of each sugars is shown above traces. Values represent the mean ± SD of three independent measurements. Blanked arrow indicates Hs Tas1R2/Tas1R3 and filled arrow indicates Mf Tas1R2/Tas1R3 concentration which ⊿F/F is significantly higher than 0 mM.

**Figure 2:**
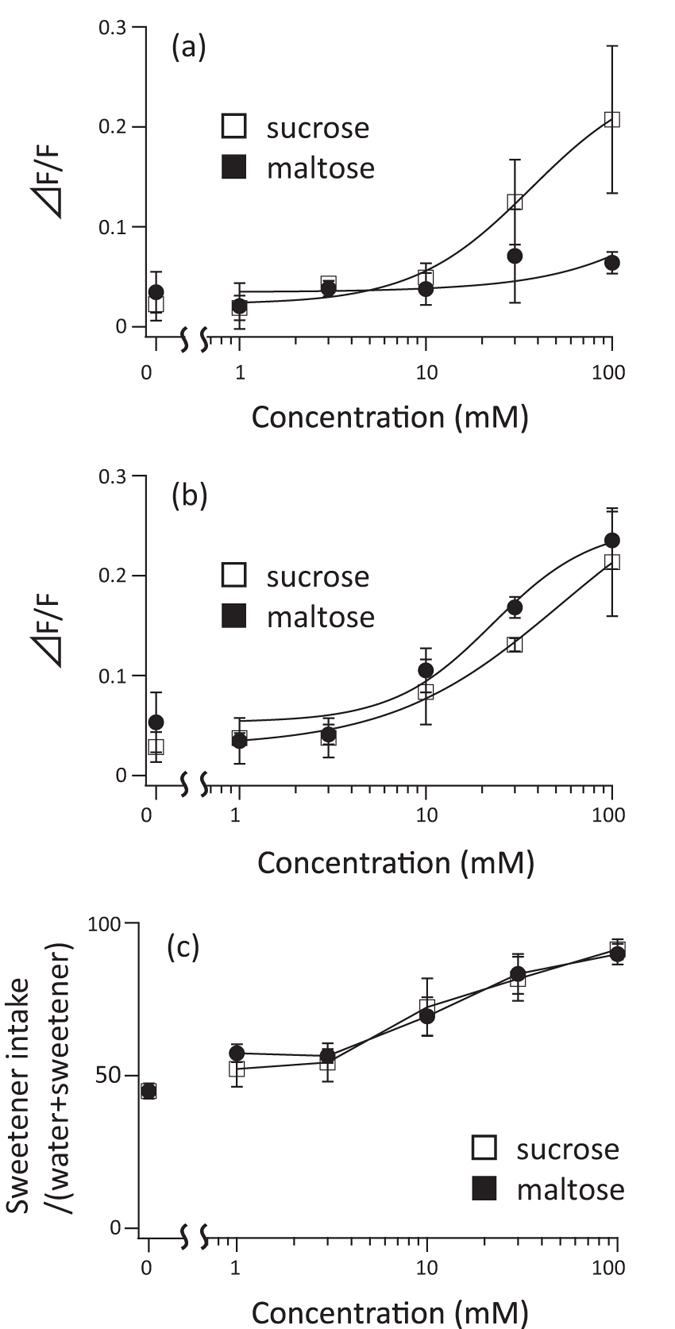
Comparison between sucrose sensitivity and maltose sensitivity. (**a**) Functional analysis of Hs Tas1R2/Tas1R3 sensitivity to sucrose (□) and maltose (■) solutions. The data correspond to those summarized in Fig. 1(a) and (b). Values represent means ± SD of three independent measurements. (**b**) Functional analysis of Mf Tas1R2/Tas1R3 to sucrose (□) and maltose (■) solutions. The data correspond to those summarized Fig. 1(a) and (b). Values represent means ± SD of three independent measurements. (**c**) Behavioral experiment of Japanese macaque responses to sucrose (□) and maltose (■) solutions. Values represent means ± SD of four individuals from four independent experiments (Supplementary Figure S1).

First, we performed an *in vitro* functional assay of Tas1R2/Tas1R3 of Japanese monkeys (Mf) and humans (Hs). HEK293T cultured cells were transfected with Tas1R2/Tas1R3 and a chimeric G protein. After cells were loaded with a calcium indicator dye, various natural sugars were added. We observed fluorescence to monitor the intracellular calcium concentration. As expected, Hs Tas1R2/Tas1R3 showed a significant dose-dependent response to sucrose and fructose^4^ (Fig. 1a,d). Surprisingly, Mf Tas1R2/Tas1R3 showed significant dose-dependent responses to maltose, sucrose, and fructose (Fig. 1a,b,d). Hs Tas1r2/Tas1R3 responded to sucrose when the concentration was higher than 10 mM, but not to maltose (Fig. 2a). Mf Tas1R2/Tas1R3 responded to sucrose and maltose, when the concentration was greater than 10 mM (Fig. 2b). This result suggests that the Japanese macaque is equally sensitive to maltose and sucrose, and can detect sweet tastes in >10 mM solutions.

To confirm whether sweet taste sensitivity in monkeys is directly related to Tas1R2/Tas1R3 function, we conducted a two-bottle behavioral experiment using four Japanese macaques. Most monkeys chose sucrose or maltose significantly more often than water when the concentration was greater than 10 mM (Figure S1). The average sweetener intakes of monkeys are shown in Fig. 2c. These thresholds were 10–30 mM, similar to the results of the Tas1R2/Tas1R3 functional assay, suggesting the contribution of Tas1R2/Tas1R3. Because the maltose sensitivities of Tas1R2/Tas1R3 in humans were very weak, these results suggest that monkey Tas1R2/Tas1R3 evolved sensitivity to maltose in addition to sucrose.

**Figure 3:**
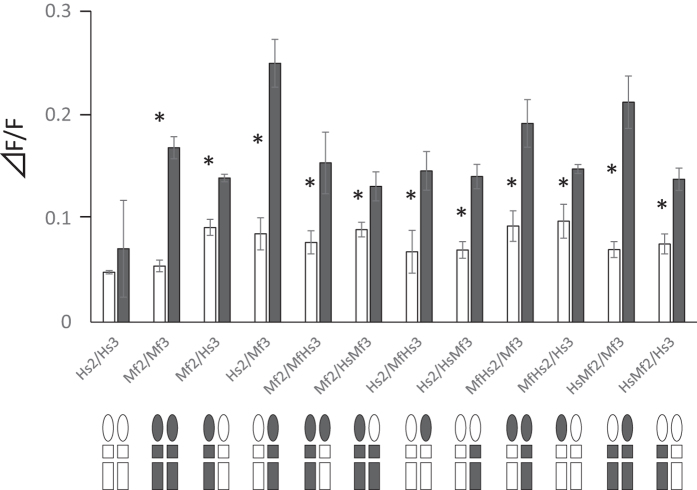
Comparisons of response of chimeric Tas1R2/Tas1R3. Responses to 0 mM (white bar) and 30 mM (black bar) maltose are shown. Bottom figures show the Tas1R2 and Tas1R3 images: Circles indicate the VFTD and squares indicate the CRD (small) and TMD (large). The Hs sequence is shown in white and the Mf sequence is shown in black. Values represent means ± SD of three independent measurements. Asterisks (*) indicate when the response to 30 mM maltose was significantly (*p* < 0.05, original values are shown in Table S1) higher than those to 0 mM maltose.

We then conducted a functional assay using chimeric Tas1R2/Tas1R3 between human and macaques to identify maltose binding site(s) (Fig. 3). We first measured the responses of Hs Tas1R2/Tas1R3 in which one side of subunit was changed to that of Japanese macaques. Although the Hs Tas1R2/Mf Tas1r3 response was greater than that of Mf Tas1R2/Hs Tas1R3, both chimeras responded to maltose. Next, we measured the responses of the Mf T1R2 and Mf T1R3 chimeras to determine the precise part of Mf Tas1Rs that is critical. All cells with co-expression of the chimera responded to 30 mM maltose. These results might suggest that all parts of Mf Tas1R2 and Mf Tas1R3 are involved in the response to maltose. Even though it is difficult to generate site-directed mutants for each subunit, this finding suggests that Tas1R3 as well as Tas1R2 are involved in the evolution of sweet taste receptors.

## Discussion

In the experiment, human and Japanese macaque Tas1R2/tas1R3 showed high responses to sucrose, as expected. Some amino acids critically related to sucrose binding are found in Hs Tas1R2. In Mf Tas1R2, amino acids at these positions correspond to those of Hs Tas1R2, except at position 40, which is serine in humans and threonine in Japanese macaques. Because both Mf Tas1R2/Tas1R3 and Hs Tas1R2/Tas1R3 responded to sucrose dose-dependently for concentrations of at least 10 mM, S40T seems to have no effect to sucrose binding to Tas1R2/Tas1R3.

Maltose is a natural sugar constructed from two glucose molecules bound with α(1–4) glycosidic bonds. This sugar is produced from starch digested by α-amylase, an enzyme secreted from the salivary gland and pancreas. Pigtail macaques (*Macaca nemestrina*) and olive baboons (*Papio anubis*) also have higher sensitivity to maltose than humans^3^. Interestingly, only these Old World monkeys had similarly high sensitivity to maltose as sucrose^3^, while other primate species did not, including the spider monkey^6^ and squirrel monkey^7^. Based on short-term behavioral experiments, mice^8^ and rats^9^ have maltose thresholds of 20 to 30 mM. Therefore, the Old World monkey lineage specifically gained high sensitivity to maltose.

Old World monkeys are general herbivores with food repertoires that extend to leaves, fruits, and seeds. Japanese macaques spend half of their annual feeding time eating leaves and seeds^10^, which contain many starches^11^. Additionally, only Old World monkeys have a cheek pouch in which they can store food items; these food items are exposed to saliva containing α-amylase for extended durations. In macaques, the levels of saliva amylase are similar to those in humans^12^, though the mechanism explaining amylase levels is unknown. Therefore, sensitivity to starches is higher in macaques than other animals, suggesting an ecological advantage for starch consumption in macaques.

Primate species have various mutations in *Tas1R2* and *Tas1R3*^13^. Generally, most sweet taste compounds bind to a huge external membrane domain called the venus flytrap domain (VFTD) of Tas1R2^14^. Some amino acid residues are critical for sucrose binding, but there is no information for maltose. The Mf Tas1R2 response to sucrose was similar to that of Hs Tas1R2, but differed from the response to maltose. In addition, these results suggested that the maltose response is related to both Tas1R2 and Tas1R3, indicating that the critical amino acids for binding are different from those for sucrose. In comparisons of Tas1R2 and Tas1R3 VFTD between Hominidae and Cercopithecinae, mutations at 45 and 43 amino acid residues have been observed, respectively^13^. We infer that these mutations affect sweet taste sensitivity to natural sugars by changing Tas1R2/Tas1R3 function. Characterizing Tas1R2/Tas1R3 function and sweet taste sensitivity may provide a basis for understanding Tas1R2/Tas1R3 evolution in primates and feeding habitat adaptations. It is possible that Tas1R2 and Tas1R3 of macaques evolved to bind to maltose, while the ability to bind to sucrose was maintained in Tas1R2. The binding of the Tas1R3 VFTD to sugars has been reported in the hummingbird^15^. In particular, hummingbird Tas1r2 was lost in the ancestor of the birds and Tas1R1/Tas1R3 became sweet receptors via a Tas1R3 binding subunit. Adding to the central region of the VFTD, amino acids at the edge of one subdomain were mutated in hummingbirds, causing Tas1R3 to function as a sweet taste receptor. In our case, chimeric Tas1R3 containing the VFTD and/or the cysteine-rich domain (CRD) plus the trans-membrane domain (TMD) of macaques exhibited a response to maltose. We speculate that not only the VFTD but also the CRD and/or TMD are effective for the maltose response, such as via conformational changes after maltose binding to Tas1R2/Tas1R3. Accordingly, Tas1R3 in animals may not only support the function of Tas1R1 or Tas1R2, but may also have the capacity to bind to sugars.

## Materials and Methods

### Sweet taste compounds

The natural sugars maltose (Wako, Osaka, Japan) and sucrose (Wako) were used for sweet taste solutions. Each compound was dissolved in deionized water to make 1, 3, 10, 30, and 100 mM solutions for the behavioral tests. To prevent potential osmotic effects, we used a maximum concentration of 100 mM. For the *in vitro* functional analysis, the compounds were dissolved in assay buffer (10 mM HEPES, 130 mM NaCl, 10 mM glucose, 5 mM KCl, 2 mM CaCl_2_, 1.2 mM MgCl_2_, pH 7.4).

### Cell culture

HEK293T cells were provided by Dr. Matsunamiani (Duke University) via Dr. Misaka (Tokyo University) for the functional analysis. Cells were cultivated in a 5% CO_2_ incubator at 37 °C with low-glucose DMEM (Sigma-Aldrich Japan, Tokyo, Japan) supplemented with 10% FBS.

### DNA

To obtain Japanese macaque (Mf) Tas1R2/Tas1R3, RNA was extracted from Japanese macaque tongues stored at the institute. RT-PCR was conducted to obtain Tas1R2 and Tas1R3 cDNA. Amplified Tas1R2 and Tas1R3 sequences were checked by sequencing using the ABI 3130xl. Human (Hs) Tas1R2 and Tas1R3 were purchased from Kazusa DNA Res. Inst. (FHC07060 and FHC10750). Tas1R2 and Tas1R3 were inserted into the mammalian expression vector pEAK10 (Edge BioSystems, Inc., Gaithersburg, MD, USA) and transfected to HEK293T cells with Gα16-gust44^16^ using Lipofectamine 3000 (Life Technologies, Inc., Carlsbad, CA, USA). Chimeric receptors were made using In-fusion kits (Takara Bio Inc., Shiga, Japan). For the functional analysis, Calcium 4 (Molecular Devices, Inc., Eugene, OR, USA) was used as an intracellular Ca^2+^ indicator. Fluorescence was measured at 525 nm following excitation at 485 nm using the FlexStation 3 Microplate Reader (Molecular Devices Japan, Inc., Tokyo, Japan). The calcium response amplitudes were expressed as ΔF/F, which is the ratio of the ligand-dependent increase in fluorescence to the fluorescence before ligand addition. The response of cells that were transfected with the empty pEAK10 vector and Gα16gust44 was defined as the mock response (TAS2R-independent response) and subtracted from all responses. ΔF/F values were fitted to the Hill equation (*y* = (max-min)/(1 + (*x*/EC50)rate)). Comparisons across concentrations were made by one-way ANOVA followed by Welch’s tests (*p* < 0.05). Dunnett’s tests were performed to determine the concentration at which significantly higher responses were observed compared to that at 0 mM (*p* < 0.05).

### Behavioral experiment

This experiment was approved by the Animal Welfare and Animal Care Committee of Primate Research Institute, Kyoto University (Permit Numbers: 2015-081 and 2016-113), based on the Guidelines for Care and Use of Nonhuman Primates of the Primates Research Institute, Kyoto University (Version 3, issued in 2010). These guidelines were prepared based on the provisions of the Guidelines for Proper Conduct of Animal Experiments (June 1, 2006; Science Council of Japan), Basic Policies for the Conduct of Animal Experiments in Research Institutions under the Jurisdiction of the Ministry of Health, Labor and Welfare (effective on June 1, 2006; Ministry of Health, Labor and Welfare (MHLW)), Fundamental Guidelines for Proper Conduct of Animal Experiment and Related Activities in Academic Research Institutions (Notice No. 71 of the Ministry of Education, Culture, Sports, Science and Technology (MEXT) dated June 1, 2006), and Standards Relating to the Care and Management of Laboratory Animals and Relief of Pain (Notice No. 88 of the Ministry of the Environment dated April 28, 2006).

Four 4–6-year-old Japanese macaques at the Primate Research Institute, Kyoto University were used. Monkeys were kept in individual cages in the same room. Access to food and water was not restricted. To measure the sweet taste threshold of monkeys, the two-bottle test^3^ was conducted. In this test, one of the bottles contained water and another contained the sweet taste solution (at various concentrations). In a trial, monkeys were exposed to two bottles at the same time for 1 min and solution intake was measured. This experiment was conducted twice a day, 1 h after feeding. The position of bottles was changed in each experiment. Four trials were performed for each concentration. The intake rates of sweet taste solutions were calculated as follows: Intake rate of sweet taste solution = Intake of sweet taste solution/Total intake. The order of exposure to solutions was randomized, except in the first trial, in which 100 mM solutions were used to motivate monkeys. A threshold concentration was calculated for each monkey according to previous methods^3^ as the lowest concentration for which a significant increase in sugar water drinking compared with a sugar concentration of 0 was detected (*p* < 0.05).

## Additional Information

**How to cite this article**: Nishi, E. *et al*. High maltose sensitivity of sweet taste receptors in the Japanese macaque (*Macaca fuscata*). *Sci. Rep.* **6**, 39352; doi: 10.1038/srep39352 (2016).

**Publisher’s note:** Springer Nature remains neutral with regard to jurisdictional claims in published maps and institutional affiliations.

## Supplementary Material

Supplementary FigureS1 and Tables
