# Supplementary material for: High maltose sensitivity of sweet taste receptors in the Japanese macaque (Macaca fuscata)
Source: Sci Rep. 2016 Dec 16;6:39352. doi: 10.1038/srep39352 (PMC5159886; doi:10.1038/srep39352)
Supplement: Supplementary FigureS1 and Tables [file srep39352-s1.pdf]

High maltose sensitivity of sweet taste receptors in the Japanese macaque (*Macaca fuscata*)

Emiko Nishi, Kei Tsutsui, and Hiroo Imai*

Supplementary Information

Additional Supporting information may be found in the online version of this article:

Figure S1. Two-bottle experiment to Japanese macaques.

Table S1. Summary of the statistical analysis of the data presented in Figure 1

Table S2. Summary of the statistical analysis of the data presented in Figure 3

Table S3. Summary of the statistical analysis of the data presented in Fig. S1

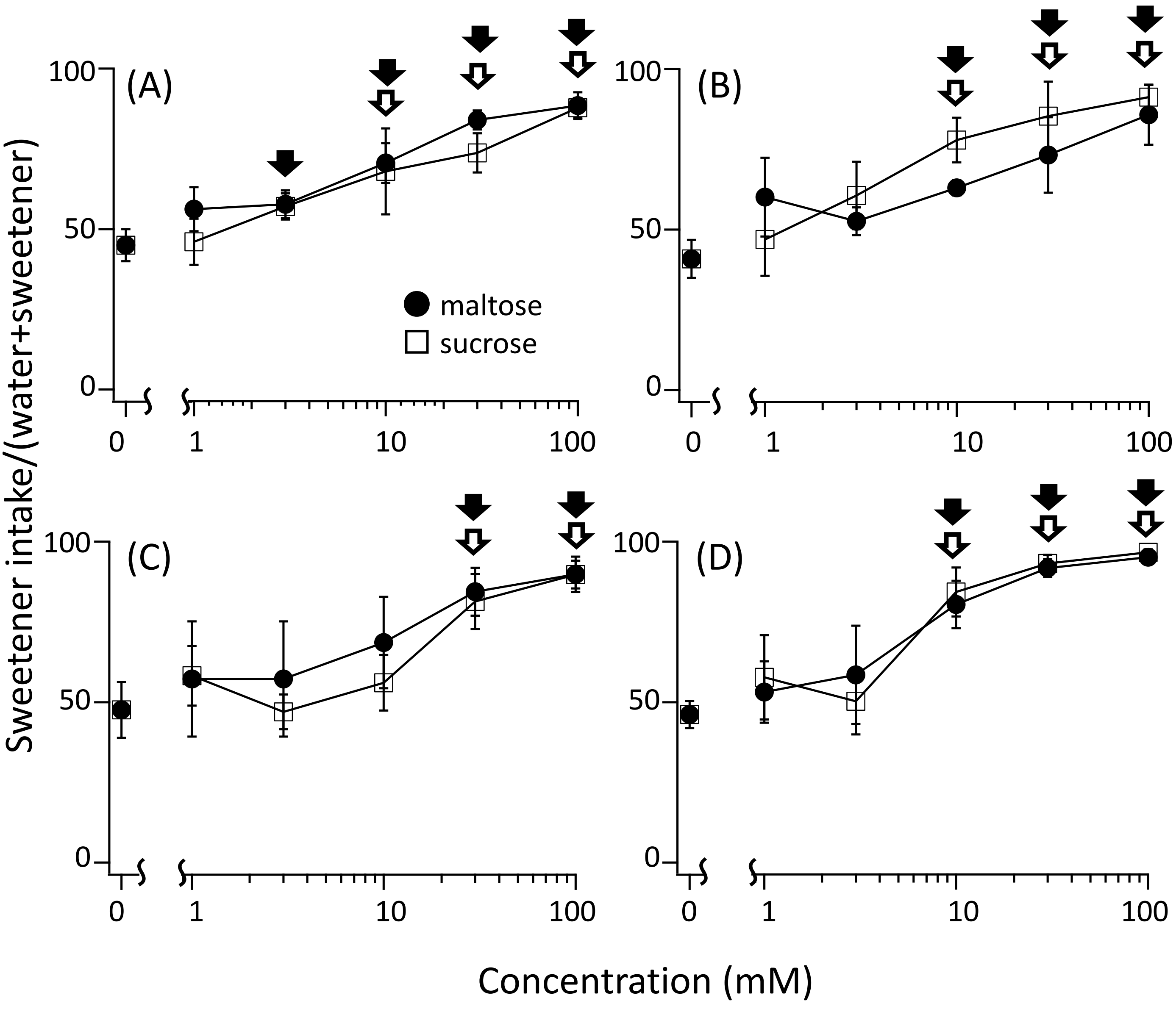

Fig. S1 Two-bottle experiment to Japanese macaques.
The experiment was conducted to four individuals (A-D). Values represent the mean $\pm$ SD of four trials. Blanked arrow indicates the sucrose and filled arrow indicates the maltose concentration which average intake rates were significantly higher than 0 mM.

Table S1: Summary of the statistical analysis of the data presented in Figure 1

sucrose n=3
humans conc.

0	1	3	10	30	100
0.01618	0.015926	0.046433	0.069522	0.172202	0.305074
0.006196	0.005707	0.040108	0.036926	0.132936	0.189888
0.04515	0.03531	0.042811	0.041571	0.069172	0.127112

one-way ANOVA

	DF	SS	MS	F	Fc	P
Groups	5	0.082563	0.016513			
Error	12	0.023614	0.001968			
Total	17	0.106176	0.006246	8.391396	3.105875	0.001293

Welch

N1	5
N2	4
Fp	1.202385
Fpc	6.256057
Pp	0.441515

Dunnett

	difference	SE	q	qp(0.05, 12, 6)	conclusion	P
0_vs_1	0.003528	0.036	0.097406	2.901257	1	0.999995
0_vs_2	-0.02061	0.036	-0.568985	2.901257	1	0.966673
0_vs_3	-0.02683	0.036	-0.740785	2.901257	1	0.910996
0_vs_4	-0.10226	0.03622	-2.823356	2.901257	1	0.057304
0_vs_5	-0.18485	0.03622	-5.103547	2.901257	0	0.001103

maltose n=3
humans conc.

0	1	3	10	30	100
0.014139	0.052667	0.040358	0.056417	0.136084	0.072609
0.062812	0.009307	0.027795	0.017477	0.029573	0.07073
0.027141	0.0002	0.046539	0.03937	0.046879	0.048765

one-way ANOVA

	DF	SS	MS	F	Fc	P
Groups	5	0.005465	0.001093			
Error	12	0.010672	0.000889			
Total	17	0.016137	0.000949	1.229067	3.105875	0.354214

Welch

N1	5
N2	4
Fp	0.483254
Fpc	6.256057
Pp	0.777337

Dunnett

	difference	SE	q	qp(0.05, 12, 6)	conclusion	P
0_vs_1	0.013973	0.024349	0.573849	2.901257	1	0.965531
0_vs_3	-0.00306	0.024349	-0.12556	2.901257	1	0.999983
0_vs_2	-0.00353	0.024349	-0.14512	2.901257	1	0.999939
0_vs_5	-0.02934	0.024349	-1.20485	2.901257	1	0.64344
0_vs_4	-0.03615	0.024349	-1.48456	2.901257	1	0.467568

glucose n=3
humans conc.

0	1	3	10	30	100
0.061965	0.054087	0.077653	0.051623	0.094422	0.132373
0.05245	0.036517	0.046104	0.078137	0.079032	0.076192
0.042173	0.019594	0.001202	0.046636	0.029976	0.047715

	DF	SS	MS	F	Fc	P
	SS	MS	F	FC	P	
Groups	5	0.004792	0.000958			
Error	12	0.010293	0.000858			
Total	17	0.015086	0.000887	1.117413	3.105875	0.40172

Welch

N1	5
N2	4
Fp	0.234243
Fpc	6.256057
Pp	0.92838

Dunnett

	difference	SE	q	qp(0.05, 12, 6)	conclusion	P
0_vs_1	0.015464	0.023913	0.646652	2.901257	1	0.945422
0_vs_2	0.010543	0.023913	0.440887	2.901257	1	0.988464
0_vs_3	-0.0066	0.023913	-0.27612	2.901257	1	0.998626
0_vs_4	-0.01561	0.023913	-0.65293	2.901257	1	0.943422
0_vs_5	-0.03323	0.023913	-1.38962	2.901257	1	0.524957

sucrose n=3
monkeys conc.

0	1	3	10	30	100
0.017323	0.037125	0.011753	0.079959	0.132854	0.245389
0.049908	0.031715	0.05904	0.1254	0.137579	0.257526
0.018737	0.044008	0.042488	0.045631	0.122083	0.137255

one-way ANOVA

	DF	SS	MS	F	Fc	P
Groups	5	0.078475	0.015695			
Error	12	0.014003	0.001167			
Total	17	0.092478	0.00544	13.45009	3.105875	0.000143

Welch

N1	5
N2	4
Fp	12.46544
Fpc	6.256057
Pp	0.014963

Dunnett

	difference	SE	q	qp(0.05, 12, 6)	conclusion	P
0_vs_1	-0.00896	0.027891	-0.321251	2.901257	1	0.997214
0_vs_2	-0.0091	0.027891	-0.326422	2.901257	1	0.997001
0_vs_3	-0.05501	0.027891	-1.972176	2.901257	1	0.235524
0_vs_4	-0.10218	0.027891	-3.663564	2.901257	0	0.012966
0_vs_5	-0.18473	0.027891	-6.623307	2.901257	0	0.000108

maltose n=3
monkeys conc.

0	1	3	10	30	100
0.048437	0.041977	0.036886	0.136247	0.182123	0.234561
0.09222	0.003567	0.03191	0.088906	0.166166	0.270661
0.019572	0.058149	0.054825	0.090311	0.156371	0.20041

one-way ANOVA

	DF	SS	MS	F	Fc	P
Groups	5	0.097902	0.01958			
Error	12	0.008796	0.000733			
Total	17	0.106697	0.006276	26.71332	3.105875	4.12E-06

Welch

N1	5
N2	4
Fp	9.323735
Fpc	6.256057
Pp	0.025193

Dunnett

	difference	SE	q	qp(0.05, 12, 6)	conclusion	P
0_vs_1	0.018846	0.022105	0.852528	2.901257	1	0.858646
0_vs_2	0.012203	0.022105	0.552022	2.901257	1	0.970467
0_vs_3	-0.05175	0.022105	-2.34082	2.901257	1	0.130618
0_vs_4	-0.11481	0.022105	-5.19375	2.901257	0	0.000952
0_vs_5	-0.1818	0.022105	-8.22424	2.901257	0	1.42E-05

glucose n=3
monkeys conc.

0	1	3	10	30	100
0.076905	0.055975	0.073521	0.066123	0.051219	0.115371
0.06785	0.054304	0.046333	0.052167	0.073453	0.1222
0.031035	0.034406	0.02618	0.038232	0.04381	0.121676

	DF	SS	MS	F	Fc	P
	SS	MS	F	FC	P	
Groups	5	0.011466	0.002293			
Error	12	0.003491	0.000291			
Total	17	0.014957	0.00088	7.882405	3.105875	0.001697

Welch

N1	5
N2	4
Fp	9.045835
Fpc	6.256057
Pp	0.026573

Dunnett

	difference	SE	q	qp(0.05, 12, 6)	conclusion	P
0_vs_1	0.010368	0.013927	0.744508	2.901257	1	0.909443
0_vs_2	0.009919	0.013927	0.712221	2.901257	1	0.922431
0_vs_3	0.006422	0.013927	0.46116	2.901257	1	0.986002
0_vs_4	0.002436	0.013927	0.174916	2.901257	1	0.999848
0_vs_5	-0.06115	0.013927	-4.39107	2.901257	0	0.003639

fructose	n=3					
humans	conc.					
	0	1	3	10	30	100
	0.036917	0.061897	0.059812	0.098736	0.12566	0.184689
	0.056992	0.044507	0.082892	0.067417	0.078558	0.179506
	0.048126	0.080193	0.089832	0.067197	0.157644	0.170154

one-way ANOVA

	DF	SS	MS	F	Fc	P
Groups	5	0.034521	0.006904			
Error	12	0.005266	0.000439			
Total	17	0.039787	0.00234	15.73361	3.105875	6.58E-05

Welch

N1	5
N2	4
Fp	17.45966
Fpc	6.256057
Pp	0.008033

Dunnett

	difference	SE	q	qp(0.05, 12, 6)	conclusion	P
0_vs_1	-0.01485	0.017104	-0.86844	2.901257	1	0.850302
0_vs_2	-0.03017	0.017104	-1.76374	2.901257	1	0.320891
0_vs_3	-0.03044	0.017104	-1.77957	2.901257	1	0.313676
0_vs_4	-0.07328	0.017104	-4.28413	2.901257	0	0.004374
0_vs_5	-0.13077	0.017104	-7.64565	2.901257	0	2.79E-05

sorbitol	n=2					
humans	conc.					
	0	1	3	10	30	100
	0.050194	0.048866	0.062368	0.069169	0.11758	0.080156
	0.045452	0.032603	0.042215	0.071201	0.051804	0.072485

one-way ANOVA

	DF	SS	MS	F	Fc	P
Groups	5	0.003069	0.000614			
Error	6	0.002541	0.000424			
Total	11	0.00561	0.00051	1.449169	4.387374	0.329372

Welch

N1	5
N2	2
Fp	4.385875
Fpc	19.29641
Pp	0.196035

Dunnett

	difference	SE	q	qp(0.05, 12, 6)	conclusion	P
0_vs_1	0.007088	0.02058	0.344407	3.388394	1	0.995428
0_vs_2	-0.00447	0.02058	-0.21715	3.388394	1	0.999462
0_vs_3	-0.02236	0.02058	-1.0866	3.388394	1	0.725471
0_vs_5	-0.0285	0.02058	-1.38473	3.388394	1	0.551095
0_vs_4	-0.03687	0.02058	-1.7915	3.388394	1	0.351584

lactose	n=2					
humans	conc.					
	0	1	3	10	30	100
	0.046809	0.085391	0.025143	0.069207	0.107204	0.098252
	0.055848	0.035665	0.035732	0.068436	0.063027	0.084435

one-way ANOVA

	DF	SS	MS	F	Fc	P
Groups	5	0.005027	0.001005			
Error	6	0.002405	0.000401			
Total	11	0.007432	0.000676	2.508667	4.387374	0.146887

Welch

N1	5
N2	2
Fp	3.657803
Fpc	19.29641
Pp	0.228522

Dunnett

	difference	SE	q	qp(0.05, 12, 6)	conclusion	P
0_vs_2	0.02089	0.02002	1.043474	3.388394	1	0.750715
0_vs_1	-0.0092	0.02002	-0.45952	3.388394	1	0.984144
0_vs_3	-0.01749	0.02002	-0.87377	3.388394	1	0.844314
0_vs_4	-0.03379	0.02002	-1.68767	3.388394	1	0.396547
0_vs_5	-0.04002	0.02002	-1.99877	3.388394	1	0.274444

fructose	n=3					
monkeys	conc.					
	0	1	3	10	30	100
	0.048711	0.078216	0.039272	0.063119	0.088825	0.126625
	0.066836	0.064025	0.053689	0.059882	0.088925	0.11845
	0.05692	0.054106	0.07377	0.069485	0.077794	0.117542

one-way ANOVA

	DF	SS	MS	F	Fc	P
Groups	5	0.009301	0.00186			
Error	12	0.001238	0.000103			
Total	17	0.010539	0.00062	18.02385	3.105875	3.29E-05

Welch

N1	5
N2	4
Fp	10.7272
Fpc	6.256057
Pp	0.019629

Dunnett

	difference	SE	q	qp(0.05, 12, 6)	conclusion	P
0_vs_2	0.001912	0.008295	0.23048	2.901257	1	0.999419
0_vs_3	-0.00667	0.008295	-0.80447	2.901257	1	0.882568
0_vs_1	-0.00796	0.008295	-0.95967	2.901257	1	0.798948
0_vs_4	-0.02769	0.008295	-3.33854	2.901257	0	0.023079
0_vs_5	-0.06338	0.008295	-7.64139	2.901257	0	2.81E-05

sorbitol	n=2					
monkeys	conc.					
	0	1	3	10	30	100
	0.023048	0.064223	0.039624	0.075554	0.124508	0.100304
	0.026919	0.038693	0.078638	0.058712	0.070684	0.111158

one-way ANOVA

	DF	SS	MS	F	Fc	P
Groups	5	0.009005	0.001801			
Error	6	0.002744	0.000457			
Total	11	0.011749	0.001068	3.938485	4.387374	0.062684

Welch

N1	5
N2	2
Fp	10.23181
Fpc	19.29641
Pp	0.09142

Dunnett

	difference	SE	q	qp(0.05, 12, 6)	conclusion	P
0_vs_1	-0.02647	0.021384	-1.23806	3.388394	1	0.635737
0_vs_2	-0.03415	0.021384	-1.59688	3.388394	1	0.439349
0_vs_3	-0.04215	0.021384	-1.97109	3.388394	1	0.283805
0_vs_4	-0.07261	0.021384	-3.39567	3.388394	0	0.049573
0_vs_5	-0.08075	0.021384	-3.77609	3.388394	0	0.031935

lactose	n=2					
monkeys	conc.					
	0	1	3	10	30	100
	0.046638	0.055974	0.05145	0.081174	0.118845	0.110797
	0.044956	0.024263	0.046581	0.015986	0.052186	0.108843

one-way ANOVA

	DF	SS	MS	F	Fc	P
Groups	5	0.007844	0.001569			
Error	6	0.004864	0.000811			
Total	11	0.012708	0.001155	1.935015	4.387374	0.222428

Welch

N1	5
N2	2
Fp	119.5222
Fpc	19.29641
Pp	0.008318

Dunnett

	difference	SE	q	qp(0.05, 12, 6)	conclusion	P
0_vs_1	0.005679	0.028473	0.199443	3.388394	1	0.999642
0_vs_3	-0.00278	0.028473	-0.09774	3.388394	1	0.999989
0_vs_2	-0.00322	0.028473	-0.11304	3.388394	1	0.999978
0_vs_4	-0.03972	0.028473	-1.39493	3.388394	1	0.545388
0_vs_5	-0.06402	0.028473	-2.24852	3.388394	1	0.201954

Table S2: Summary of the statistical analysis of the data presented in Figure 3

Two-way ANOVA

Table of Analysis of Variance

source	SS	df	MS	F	p	
A:receptor	0.0452556	11	0.0041141	8.970	0.0000	****
B:conc	0.1176293	1	0.1176293	256.467	0.0000	****
AB	0.0294118	11	0.0026738	5.830	0.0000	****
error[WC]	0.0220153	48	0.0004587			
Total	0.2143120	71				

+ p<.10, * p<.05, ** p<.01, *** p<.005, **** p<.001

<< means for AB interaction >>

effect	SS	df	MS	F	p	
A(b1)	0.0078813	11	0.0007165	1.562	0.1411	
A(b2)	0.0667861	11	0.0060715	13.238	0.0000	****
error		48	0.0004587			
B(a1)	0.0007711	1	0.0007711	1.681	0.2010	
B(a2)	0.0194847	1	0.0194847	42.483	0.0000	****
B(a3)	0.0034038	1	0.0034038	7.421	0.0090	**
B(a4)	0.0405537	1	0.0405537	88.419	0.0000	****
B(a5)	0.0088079	1	0.0088079	19.204	0.0001	****
B(a6)	0.0026060	1	0.0026060	5.682	0.0211	*
B(a7)	0.0091362	1	0.0091362	19.920	0.0000	****
B(a8)	0.0075254	1	0.0075254	16.408	0.0002	****
B(a9)	0.0147337	1	0.0147337	32.124	0.0000	****
B(a10)	0.0037937	1	0.0037937	8.271	0.0060	**
B(a11)	0.0303659	1	0.0303659	66.207	0.0000	****
B(a12)	0.0058590	1	0.0058590	12.774	0.0008	****
error		48	0.0004587			

+ p<.10, * p<.05, ** p<.01, *** p<.005, **** p<.001

Table S3: Summary of the statistical analysis of the data presented in Fig. S1

(A)

maltose	0	1	3	10	30	100
	50	50	50	75	83.33333	86.36364
	40	57.14286	60	71.42857	80	86.66667
	50	66.66667	60	80	88.23529	84.61538
	40	50	60	60	83.33333	95.2381

one-way ANOVA

	DF	SS	MS	F	Fc	P
Groups	5	5790.385	1158.077			
Error	18	681.415	37.85639			
Total	23	6471.8	281.3826	30.59132	2.772853	3.38E-08

Welch

N1	5
N2	7
Fp	8.388502
Fpc	3.971523
Pp	0.007197

Dunnett

	difference	SE	q	qp(0.05, 12, 6)	conclusion	P
0_vs_1	-10.9524	4.350655	-2.51741	2.761412	1	0.080877
0_vs_2	-12.5	4.350655	-2.87313	2.761412	0	0.039883
0_vs_3	-26.6071	4.350655	-6.11566	2.761412	0	6.88E-05
0_vs_4	-38.7255	4.350655	-8.90107	2.761412	0	2.83E-05
0_vs_5	-43.2209	4.350655	-9.93435	2.761412	0	2.81E-05

sucrose	0	1	3	10	30	100
	50	33.33333	50	50	71.42857	85.71429
	40	50	60	77.77778	72.72727	84.21053
	50	50	57.14286	60	66.66667	88
	40	50	60	83.33333	83.33333	92.30769

	DF	SS	MS	F	Fc	P
Groups	5	5575.729	1115.146			
Error	18	1279.319	71.07329			
Total	23	6855.048	298.0456	15.69008	2.772853	5.14E-06

Welch

N1	5
N2	7
Fp	8.657854
Fpc	3.971523
Pp	0.006577

Dunnett

	difference	SE	q	qp(0.05, 12, 6)	conclusion	P
0_vs_1	-0.83333	5.961262	-0.13979	2.761412	1	0.999954
0_vs_2	-11.7857	5.961262	-1.97705	2.761412	1	0.21573
0_vs_3	-22.7778	5.961262	-3.82097	2.761412	0	0.005376
0_vs_4	-28.539	5.961262	-4.7874	2.761412	0	0.000685
0_vs_5	-42.5581	5.961262	-7.13911	2.761412	0	3.35E-05

(B)

maltose	0	1	3	10	30	100
	33.33333	50	60	62.5	84.21053	87.5
	40	60	50	62.5	66.66667	70
	40	50	50	66.66667	57.14286	93.33333
	50	80	50	66.66667	84.61538	92

one-way ANOVA

	DF	SS	MS	F	Fc	P
Groups	5	4926.969	985.3939			
Error	18	1733.767	96.32042			
Total	23	6660.737	289.5973	10.23037	2.772853	9.07E-05

Welch

N1	5
N2	7
Fp	2.858038
Fpc	3.971523
Pp	0.101789

Dunnett

	difference	SE	q	qp(0.05, 12, 6)	conclusion	P
0_vs_2	-11.6667	6.939756	-1.68114	2.761412	1	0.345398
0_vs_1	-19.1667	6.939756	-2.76186	2.761412	0	0.049955
0_vs_3	-23.75	6.939756	-3.42231	2.761412	0	0.012657
0_vs_4	-32.3255	6.939756	-4.65802	2.761412	0	0.000896
0_vs_5	-44.875	6.939756	-6.46637	2.761412	0	4.83E-05

sucrose	0	1	3	10	30	100
	33.33333	30	77.77778	71.42857	92.59259	96
	40	57.14286	54.54545	80	91.66667	85.18519
	40	42.85714	60	71.42857	40	91.30435
	50	57.14286	60	88.23529	90	92.30769

one-way ANOVA

	DF	SS	MS	F	Fc	P
Groups	5	7766.893	1553.379			
Error	18	3203.061	177.9479			
Total	23	10969.95	476.9545	8.729403	2.772853	0.000241

Welch

N1	5
N2	7
Fp	6.329579
Fpc	3.971523
Pp	0.015636

Dunnett

	difference	SE	q	qp(0.05, 12, 6)	conclusion	P
0_vs_1	-5.95238	9.432599	-0.63104	2.761412	1	0.951884
0_vs_2	-22.2475	9.432599	-2.35857	2.761412	1	0.109373
0_vs_3	-36.9398	9.432599	-3.91618	2.761412	0	0.004377
0_vs_4	-37.7315	9.432599	-4.00012	2.761412	0	0.003652
0_vs_5	-50.366	9.432599	-5.33956	2.761412	0	0.000231

(C)

maltose	0	1	3	10	30	100
	50	60	40	50	89.47368	81.81818
	57.14286	60	66.66667	62.5	71.42857	87.5
	33.33333	62.5	40	62.5	88.88889	95.12195
	50	57.14286	81.81818	72.72727	87.5	94.73684

one-way ANOVA

	DF	SS	MS	F	Fc	P
Groups	5	5474.124	1094.825			
Error	18	2212.819	122.9344			
Total	23	7686.943	334.2149	8.905766	2.772853	0.000214

Welch

N1	5
N2	7
Fp	4.075404
Fpc	3.971523
Pp	0.047094

Dunnett

	difference	SE	q	qp(0.05, 12, 6)	conclusion	P
0_vs_2	-9.50216	7.840101	-1.212	2.761412	1	0.634028
0_vs_1	-12.2917	7.840101	-1.56779	2.761412	1	0.407035
0_vs_3	-14.3128	7.840101	-1.82558	2.761412	1	0.276439
0_vs_4	-36.7037	7.840101	-4.68154	2.761412	0	0.000853
0_vs_5	-42.1752	7.840101	-5.37942	2.761412	0	0.000214

sucrose	0	1	3	10	30	100
	50	50	50	57.14286	88.23529	91.66667
	57.14286	72.72727	50	41.66667	84.61538	82.35294
	33.33333	60	37.5	77.77778	85.71429	93.75
	50	50	50	61.53846	66.66667	90.90909

one-way ANOVA

	DF	SS	MS	F	Fc	P
Groups	6	21230.47	3538.412			
Error	22	1828.573	83.11696			
Total	28	23059.04	823.5373	42.57147	2.549061	5.23E-11

Welch

N1	6
N2	8
Fp	34.72569
Fpc	3.58058
Pp	2.64E-05

Dunnett

	difference	SE	q	qp(0.05, 12, 6)	conclusion	P
0_vs_2	0.744048	7.075585	0.105157	2.761412	1	0.999994
0_vs_1	-10.5628	7.075585	-1.49285	2.761412	1	0.451186
0_vs_3	-11.9124	7.075585	-1.68359	2.761412	1	0.344135
0_vs_4	-33.6889	7.075585	-4.76128	2.761412	0	0.000723
0_vs_5	-42.0506	7.075585	-5.94306	2.761412	0	8.60E-05

(D)

maltose	0	1	3	10	30	100
	44.44444	66.66667	42.85714	70	86.95652	93.87755
	50	57.14286	57.14286	76.47059	92.30769	96.49123
	40	42.85714	50	83.33333	94.44444	96.2963
	50	45.45455	83.33333	86.95652	92.68293	93.22034

one-way ANOVA

	DF	SS	MS	F	Fc	P
Groups	5	8670.053	1734.011			
Error	18	1578.485	87.69361			
Total	23	10248.54	445.5886	19.77351	2.772853	9.62E-07

Welch

N1	5
N2	7
Fp	15.54029
Fpc	3.971523
Pp	0.00114

Dunnett

	difference	SE	q	qp(0.05, 12, 6)	conclusion	P
0_vs_1	-6.91919	6.621692	-1.04493	2.761412	1	0.745711
0_vs_2	-12.2222	6.621692	-1.84579	2.761412	1	0.267665
0_vs_3	-33.079	6.621692	-4.99555	2.761412	0	0.000449
0_vs_4	-45.4868	6.621692	-6.86936	2.761412	0	3.73E-05
0_vs_5	-48.8602	6.621692	-7.37882	2.761412	0	3.15E-05

sucrose	0	1	3	10	30	100
	44.44444	60	33.33333	73.33333	95.34884	98.11321
	50	36.84211	60	80.95238	95.65217	96.22642
	40	60	57.14286	89.47368	92.5	95.91837
	50	73.33333	50	92.85714	88.88889	96

one-way ANOVA

	DF	SS	MS	F	Fc	P
Groups	5	10203.96	2040.792			
Error	18	1453.341	80.74119			
Total	23	11657.3	506.8393	25.27573	2.772853	1.50E-07

Welch

N1	5
N2	7
Fp	18.00437
Fpc	3.971523
Pp	0.000718

Dunnett

	difference	SE	q	qp(0.05, 12, 6)	conclusion	P
0_vs_2	-4.00794	6.353786	-0.6308	2.761412	1	0.951957
0_vs_1	-11.4327	6.353786	-1.79936	2.761412	1	0.288147
0_vs_3	-38.043	6.353786	-5.98746	2.761412	0	8.10E-05
0_vs_4	-46.9864	6.353786	-7.39502	2.761412	0	3.14E-05
0_vs_5	-50.4534	6.353786	-7.94068	2.761412	0	2.93E-05
